# Insect *in vitro* System for Toxicology Studies — Current and Future Perspectives

**DOI:** 10.3389/ftox.2021.671600

**Published:** 2021-07-19

**Authors:** Sheeja Cc, Damodaran Arun, Lekha Divya

**Affiliations:** Department of Zoology, Central University of Kerala, Kasaragod, India

**Keywords:** *in vitro* cell culture, toxicology, invertebrate, insect, toxicity screening

## Abstract

*In vitro* cell culture practices are valuable techniques to understand the mechanisms behind vital *in vivo* biological processes. *In vitro* cells have helped us to attain a deeper understanding of functions and mechanisms conserved in the course of evolution. Toxicology studies are inevitable in drug discovery, pesticide development, and many other fields that directly interact with human beings. The proper involvement and regulatory steps that have been taken by animal ethical societies in different parts of the world resulted in the reduced *in vivo* use of mammals in toxicological studies. Nevertheless, experimental animals are being killed where no replacement is available. The use of mammals could be reduced by using the *in vitro* systems. Nowadays, invertebrate cell lines are also play important role in toxicology testing. This review analyzes the cause and consequence of insect *in vitro* models in toxicology studies.

## Introduction

Across centuries, research involving animals has contributed significantly to the progress of biomedical sciences (Festing and Wilkinson, 2007). However, use of animals in research experiments became a subject of controversy. The pain and sufferings experienced by animals had arisen the moral concerns which led to anti-vivisection movement. In 1824, the Royal Society for the Prevention of Cruelty to Animals^1^, one of the oldest animal welfare charities was formed. Later in 1876, an act for preventing cruelty to the animals was made in the UK (Balls, 1994; Doke and Dhawale, 2015). In India, parellel movements were initiated in the year 1960 (Doke and Dhawale, 2015). Then, the Animals Scientific Procedures Act (1986) came into existence and provided effective regulations enforced by law. During all these progressions, 3 Rs—replacement, reduction, and refinement of animals' use in research (Russell and Burch, 1959) have been proposed. The idea of “replacement” has led the researchers to search for alternatives. It mainly relied on replacing experimental mammals like rabbits, rats, and pigs with non-mammalian systems. Invertebrate models and micro-organisms were also introduced as a replacement to vertebrate models. Most of the intriguing biological questions could be addressed using lower group of organisms. In this review, we highlight the transformative potential of insect invertebrate system for *in vitro* toxicology studies.

## Relevance of Invertebrates in Research

Invertebrates have made significant contributions in biomedical research as most of their organs and their physiology are analogous to mammals (Svidersky and Plotnikova, 2002; Roch et al., 2014). Biomedical research using invertebrates has appeared in the scientific literature from the 1900's (Wilson-Sanders, 2011). They range from terrestrial invertebrates to freshwater and marine beings, including crustaceans and mollusks. During the last decade, articles published in PubMed revealed more than 250,000 entries on invertebrate models, which is comparatively higher than the previous year reports (177,000). This indicates the efficiency of invertebrates as models in the biomedical research. Significant discoveries were made using invertebrates ranging from embryonic development to aging. For example, *Caenorhabditis elegans*, a nematode, possesses several advantages over the vertebrate model for biomedical research. For example, it has served as a model for the study on Parkinson's, Alzheimer's, Huntington's diseases, diabetes, cancer, immune disorders, and the development of therapeutic agents for these diseases (Faber et al., 1999; Link, 2001; Artal-Sanz et al., 2006; Nass et al., 2008; Pujol et al., 2008) having short generation time and inexpensive maintenance as its advantages (Nass et al., 2008). Other invertebrates have also been studied; for example, research on the squid giant axon provided the basis for the iconic nature of the electrical action potential in nerve transmission (Hodgkin and Huxley, 1952). In addition, invertebrates can be used for some screening purposes because their nervous systems are sufficiently complex and biochemically related to the human nervous system (https://www.ncbi.nlm.nih.gov/books/NBK218269/) (National Research Council (US) and Institute of Medicine (US) Committee on the Use of Laboratory Animals in Biomedical and Behavioral Research et al., 1988). Rapid shifts in ethical regulations related to vertebrate experimentation point out how dynamic these issues have been. Nowadays, ethical concerns are being raised regarding the use of invertebrates too. Animal rights organizations like PETA (2017, 2018) and social activists like Geer (2015) have raised concerns about the ethical treatment of invertebrates (Drinkwater et al., 2019).

## Insects as an *in vitro* System

Insect cells have been successfully cultured *in vitro* for over 55 years. The first-ever invertebrate tissue culture started in 1915 when Richard Goldschmidt tried to examine silkworm testes' development in culture (Goldschmidt, 1915). Later in 1935, William Trager observed that silkworm testes in culture are suitable for virus replication studies (Trager, 1935). The first continuous invertebrate cell lines were developed by Grace (1962), from ovaries of the Australian Emperor gum moth, *Antheraea eucalypti*. Later, insect cells have emerged as a cost effective alternative host platform to the mammalian cell lines. A lesser manufacturing and maintenance cost than mammalian cells could be the reason behind such a rapid change.

Most insect cells can be cultivated over a temperature range of 25–30°C and a pH of 6.2. Unlike mammalian cells, insect cell lines utilize a phosphate buffering system (Lynn, 2002). The most established insect cell lines include S2 derived from D. melanogaster, Sf9 from *Spodoptera frugiperda*, and High Five from *Trichoplusiani* (Yee et al., 2018) (Table 1). Insect cell lines have advantages like non-essentiality of CO_2_, reduced biosafety requirements (BSL1), etc. (Airenne et al., 2013).

**Table 1:** Examples for well-established insect cell lines.

**Sl. no**.	**Insect *in vitro* system**	**Major use**
1	*Aedes albopictus* C6/36	Cadmium pathology (Braeckman et al., 1999)
2	*Spodoptera fugiperda* (Sf9)	Cancer therapeutic strategies (Jahanian-Najafabadi et al., 2012)Fungal toxicity (Zhang et al., 2017)
3	*Spodoptera fugiperda* (Sf21)	Recombinant protein expression system
4	HighFive (*Trichoplusiani*)	Recombinant protein expression system
5	S2 (*D. melanogaster*)	Metal toxicity, pollutant toxicity

Among invertebrates, insects have been used as model organisms in biomedical research for a long time. The cornerstone insect widely used is *Drosophila* (Baker and Thummel, 2007). *D. melanogaster* is one of the well-studied organisms in the animal kingdom. Cytogenetic research has led to the complete mapping and sequencing of its chromosomes, leading to their continuous use in biological and biomedical investigations (Gilbert, 2008). For instance, research on the eye pigmentation of *Drosophila* led to the hypothesis that each gene controls a single enzyme—a concept that has proved fundamental to modern molecular biology (Ephrussi, 1942). Insects are well-proved as the best *in vivo* alternatives to mammals. The insect models, *D. melanogaster, Galleria mellonella, Bombyx mori*, and *Manduca sexta* provided similar results that can be obtained with mammalian systems (Fuchs and Mylonakis, 2006).

**Figure 1:**
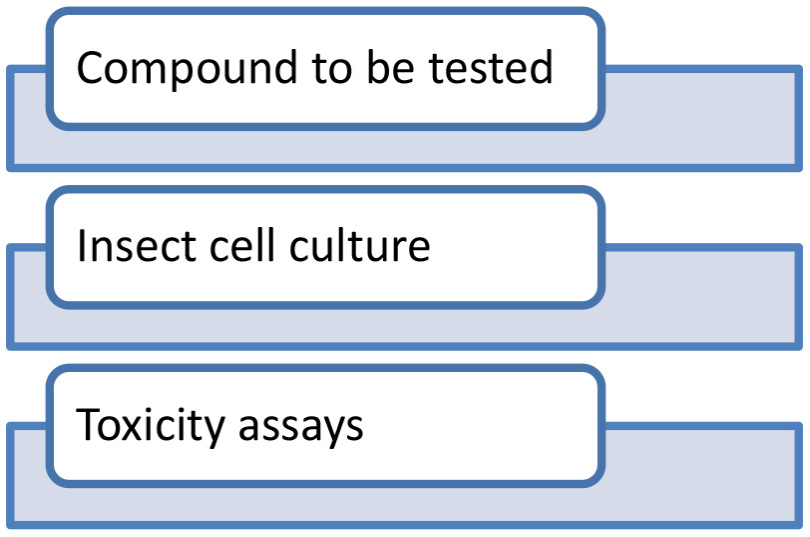
Schematic workflow of the toxicity testing using insect cell lines.

Established protocols for testing chemicals in insects are already available (Perry et al., 1998). Compounds to be tested are injected to appropriate insect cell lines and subsequent toxicity assays are performed (Figure 1). Insecticidal effects of various compounds such as actions of biflavonesin on neuronal cells (Ren et al., 2021), Bt toxicity in the cabbage looper, *Trichoplusiani* (Wang et al., 2018), and CF-203 cell line (Li et al., 2018) were studied in detail, and it provided reliable results also. *G. mellonella* has a great future in the field of toxicology and as a model for antibacterial drug testing (Cutuli et al., 2019). The structural and functional similarities with mammalian immune system make insect hemocytes a suitable platform for conducting toxicology studies at immune system level (Browne et al., 2013). The homology in proteins such as insect malvolio and dSR-C1 with the macrophage protein NRAMP-1 (Kavanagh and Reeves, 2004) in mouse makes a strong case for using insects as an alternative. Recently, Sheeja et al. (2020) studied the cellular toxicity effects of molybdenum disulfide nanoparticles in the hemocytes culture of *Oecophylla smaragdina*. Likewise, environmental monitoring studies and screening of nanoparticles were conducted on many insect species such as *D. melanogaster* (Armstrong et al., 2013; Araj et al., 2015), *Blaberus discoidalis* (Zhou et al., 2020), *B. mori* (Pandiarajan and Krishnan, 2017; Mir et al., 2020), and Honey bees (Dubey et al., 2015). Recent advances in insect baculovirus expression vector (BEV) system indicate the growing industrial interest in commercial use of the BEV/insect cell culture system for the manufacture of biopharmaceuticals. Likewise, insect cell lines have a brighter future in the development of protein therapeutics (Yee et al., 2018). Recently, a COVID-19 candidate vaccine was formulated using insect cell lines in China (Li et al., 2020). Along with advancements, there are a lot of limitations attached with insect cell line models. Though the insect cell system becomes famous for *in vitro* approaches, there are evident limitations. N- glycosylation in insect cell lines is not alike as seen in mammalian systems. Many insect species lack capabilities to elongate the core pentasaccharide Man3GlcNac2. It will be difficult if the recombinant protein require glycans for its maintenance. Likewise, “the cell density effect,” a sudden decline in the productivity and cell concentration is prominent in insect cell lines. Insect cell lines should be modified in a way that it meets all the requirements that a mammalian cell lines generally performs. Generating adequately modified insect cell lines may replace the existing mammalian cell lines.

## Conclusion

The invertebrate models especially insects are established as successful *in vivo* models for toxicological studies. That could greatly replace many mammalian systems too. Insect *in vitro* models started gaining attention considering its less maintenance time and cost-effectiveness. Many insect cell lines offer several advantages over mammalian cell lines. Detailed studies are necessary to further reveal the great potential of invertebrate, especially insect *in vitro* system.

## Author Contributions

SC, DA, and LD: wrote the manuscript. All authors contributed to the article and approved the submitted version.

## Conflict of Interest

The authors declare that the research was conducted in the absence of any commercial or financial relationships that could be construed as a potential conflict of interest. The handling editor declared a past co-authorship with several of the authors DA and LD.

## Footnotes

^1^Royal Society for the Prevention of Cruelty to the Animals. Available online at: https://www.rspca.org.uk/.
